# Circulating Omega-6, But Not Omega-3 Polyunsaturated Fatty Acids, Are Associated with Clinical Outcomes in Patients with Acute Decompensated Heart Failure

**DOI:** 10.1371/journal.pone.0165841

**Published:** 2016-11-08

**Authors:** Toshiyuki Nagai, Yasuyuki Honda, Yasuo Sugano, Kunihiro Nishimura, Michikazu Nakai, Satoshi Honda, Naotsugu Iwakami, Atsushi Okada, Yasuhide Asaumi, Takeshi Aiba, Teruo Noguchi, Kengo Kusano, Hisao Ogawa, Satoshi Yasuda, Toshihisa Anzai, NaDEF investigators

**Affiliations:** 1 Department of Cardiovascular Medicine, National Cerebral and Cardiovascular Center, Osaka, Japan; 2 Preventive Medicine and Epidemiology Informatics, National Cerebral and Cardiovascular Center, Osaka, Japan; Osaka University Graduate School of Medicine, JAPAN

## Abstract

**Background:**

Circulating polyunsaturated fatty acid (PUFA) levels are associated with clinical outcomes in cardiovascular diseases including coronary artery disease and chronic heart failure (HF). However, their clinical implications in acute decompensated HF (ADHF) remain unclear. The aim of this study was to investigate the clinical roles of circulating PUFAs in patients with ADHF.

**Methods:**

Circulating levels of PUFAs, eicosapentaenoic acid (EPA), docosahexaenoic acid (DHA), arachidonic acid (AA) and dihomo-gamma linoleic acid (DGLA), were measured on admission in 685 consecutive ADHF patients. Adverse events were defined as all-cause death and worsening HF.

**Results:**

During a median follow-up period of 560 days, 262 (38.2%) patients had adverse events. Although patients with adverse events had lower n-6 PUFA (AA + DGLA) level than those without, n-3 PUFA (EPA + DHA) level was comparable between the groups. Kaplan-Meier analyses showed that lower n-6 PUFA level on admission was significantly associated with the composite of all-cause death and worsening HF, all-cause death, cardiovascular death and worsening HF (p < 0.001, p = 0.005, p = 0.021, p = 0.019, respectively). In a multivariate Cox model, lower n-6 PUFA level was independently associated with increased risk of adverse events (HR 0.996, 95% CI: 0.993–0.999, p = 0.027).

**Conclusions:**

Lower n-6 but not n-3 PUFA level on admission was significantly related to worse clinical outcomes in ADHF patients. Measurement of circulating n-6 PUFA levels on admission might provide information for identifying high risk ADHF patients.

## Introduction

Heart failure (HF) is a common and growing public health problem worldwide. Although pharmacologic and non-pharmacologic therapies have been developed for patients with HF, mortality and morbidity remain significantly high [1, 2]. It is noteworthy that HF is a multisystem disease with multiple comorbidities including anemia, renal and hepatic dysfunction, malnutrition and cardiac cachexia [3–5]. Among them, poor nutritional status has been reported to be strongly associated with worse clinical outcomes in both acute and chronic HF patients [6–8]. Patients with HF are more prone to malnutrition for a variety of reasons, including symptomatic anorexia, early satiation, ascites, prescribed medication, psychological factors and catabolic/anabolic imbalance, which lead to a hypermetabolic state [9–11], and these are frequently observed in acute decompensated HF (ADHF) as an advanced stage of HF (stage C or D).

Polyunsaturated fatty acids (PUFAs), which are macronutrients, have usually been shown to be associated with beneficial health effects on cardiovascular disease (CVD) including coronary artery disease and HF [12, 13]; nevertheless, the two major types of long chain PUFAs, omega-3 (n-3) and omega-6 (n-6) PUFAs, were shown to have antagonistic effects. While the n-3 PUFAs emerged as anti-inflammatory and cardioprotective, the n-6 PUFAs were shown to be proinflammatory [14, 15]. Eicosanoids derived from n-3 PUFAs, such as eicosapentaenoic acid (EPA) and docosahexaenoic acid (DHA), have been shown to have anti-inflammatory and antithrombotic effects in experimental and clinical settings, whereas those derived from n-6 PUFAs, arachidonic acid (AA) and dihomo-gamma linoleic acid (DGLA), act in an opposite manner. Indeed, despite conflicting evidence [16, 17], several clinical trials have demonstrated the cardioprotective effects of n-3 PUFAs to reduce cardiovascular events, including sudden cardiac death and acute myocardial infarction (AMI), in primary and secondary prevention settings of CVD [18, 19]. Furthermore, in an epidemiological study, decreased n-3 PUFA level was also associated with worse clinical outcomes following AMI [20]. However, the association between n-6 PUFAs and CVD and the role of PUFAs in patients with ADHF remain to be determined.

Because neither n-3 nor n-6 PUFAs can be synthesized *de novo* in mammals and are considered nutritionally essential [21], we hypothesize that circulating PUFAs may objectively reflect dietary consumption and also biologically relevant processes, such as absorption, incorporation, and metabolism, and therefore impairment of their absorption in decompensated HF could relate to worse clinical outcomes.

The aim of the current study was to clarify the roles of PUFAs in the clinical setting of ADHF.

## Methods

### Study Design

Data from the National cerebral and cardiovascular center acute DEcompensated heart Failure (NaDEF) registry, obtained between January 2013 and August 2015, were retrospectively analyzed. The NaDEF registry is a single-center, observational, on-going, prospective cohort that includes all patients requiring hospitalization to our institution for the first time with a diagnosis of ADHF by at least two experienced cardiologists according to the Framingham ADHF criteria [22]. Follow-up was performed at 3, 6, 12, and 24 months after discharge by direct contact with patients or the patients’ physicians in the hospital or outpatient clinic, telephone interview of patients or, if deceased, family members, and mail, by dedicated coordinators and investigators. Currently, 92.8% of enrolled patients are successfully followed-up. In this study, because patient information was anonymized and de-identified prior to analyses, written informed consent was not obtained from each patient. However, we publicized the study by posting a summary of the protocol (with an easily understood description) on the website of the National Cerebral and Cardiovascular Center; the notice clearly informed patients of their right to refuse enrollment. These procedures for informed consent and enrollment are in accordance with the detailed regulations regarding informed consent described in the guidelines, and this study, including the procedure for enrollment, has been approved by the Institutional Review Board of the National Cerebral and Cardiovascular Center (M22-025), and registered under the Japanese UMIN Clinical Trials Registration (UMIN000017024).

### Study Population

**Fig 1:**
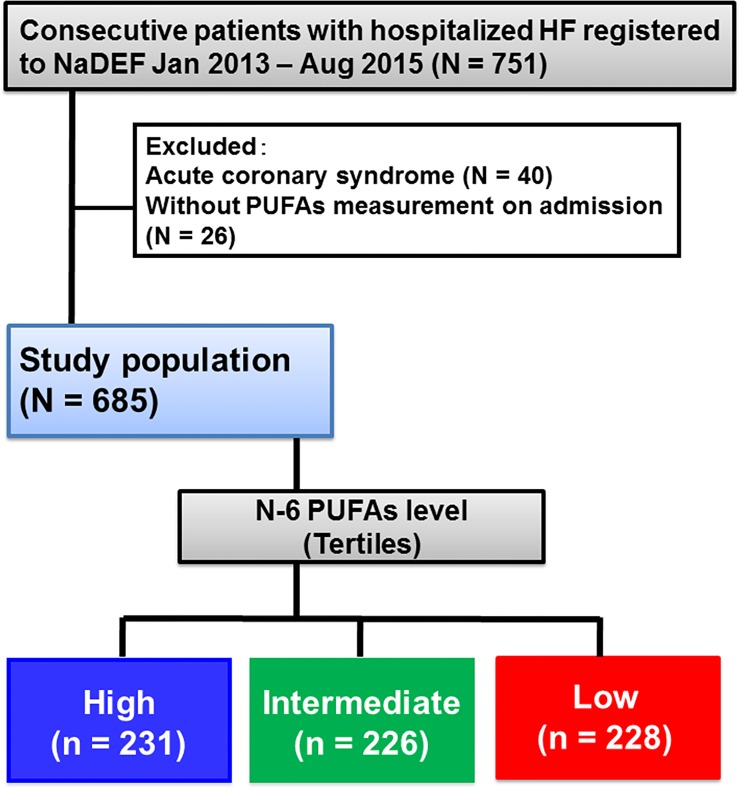
Flow diagram of study population.

From the 751 patients enrolled in the NaDEF registry, those with acute coronary syndrome receiving an EPA or DHA preparation, and those without measurement of PUFAs were excluded from this study. Finally, 685 patients were examined. Then the patients were divided into three groups according to the tertiles of n-6 PUFA level (low [< 157 μg/mL], intermediate [157–198 μg/mL], and high [199 μg/mL ≤] levels) for obtaining more detailed risk stratification on future adverse events (Fig 1).

### Blood Sampling, PUFA Measurements and Assessment of Nutritional Status

Venous blood samples were collected on admission, and routine laboratory tests were performed within a day of blood sampling using an automated analyzer, whereas additional measurements including PUFAs were performed afterward using freshly frozen samples kept at –80°C. Frozen serum samples were shipped to LSI Medience Corp., Tokyo, Japan, and EPA, DHA, AA and DGLA were measured by LC-MS/MS method, using a LC-MS8030 system (Shimadzu Corp., Kyoto, Japan). For the assessment of nutritional status, we used geriatric nutritional risk index (GNRI) which derived based on serum albumin level and body weight and has been recently validated for risk stratification in Japanese elderly ADHF patients [23].

### Study Endpoints

The study endpoints were all-cause death, cardiovascular death and worsening HF, which was defined as worsening of symptoms and signs of HF requiring intensification of intravenous therapy or initiation of mechanical support during hospitalization, as used in major ADHF clinical trials [24, 25], or readmission because of HF after discharge.

### Statistical Analyses

Results are presented as mean ± SD when normally distributed, and as median and interquartile range (IQR) when non-normally distributed. Differences between groups were analyzed by unpaired Student’s t-test, Mann-Whitney U test, or ANOVA for continuous variables, and by chi-squared test or Fisher’s exact test for dichotomous variables, when appropriate. For survival analyses, Kaplan-Meier survival plots were constructed by dividing n-6 PUFA levels on admission into tertiles (low, intermediate, and high levels), and log-rank testing was performed, to study the influence of n-6 PUFA levels on all-cause death, cardiovascular death and worsening HF. To evaluate the influence of n-6 PUFA levels on all-cause death and worsening HF, we constructed the following three Cox proportional-hazards regression models: model 1, crude; model 2, age, sex-adjusted; and model 3, fully adjusted. In model 3, based on the variables achieving p < 0.10 on univariate Cox analysis for adverse events and/or significant association with n-6 PUFA level, we included the following covariates: age, sex, ischemic cardiomyopathy etiology, systolic blood pressure, heart rate, estimated glomerular filtration rate (eGFR), GNRI, and hemoglobin, serum sodium, brain natriuretic peptide, total cholesterol, total bilirubin and C-reactive protein (CRP) levels, and history of atrial fibrillation, dyslipidemia, diabetes and chronic kidney disease, and use of statins, diuretics, spironolactone, angiotensin-converting enzyme inhibitors (ACE-Is) or angiotensin II receptor blockers (ARBs), beta blockers and intravenous inotropes. All tests were two tailed, and a value of p < 0.05 was considered statistically significant. All analyses were performed with SPSS^®^ for Windows version 23.0 (IBM, Corp., Armonk, NY, USA) and STATA^®^ 13 (Stata Corp., College Station, TX, USA).

## Results

### Baseline Characteristics and PUFA Levels in Patients With and Without Adverse Events

Of the 683 study patients, 262 (38.2%) had adverse events (all-cause death or worsening HF) during a median follow-up period of 560 days (interquartile range: 340 to 800 days). Mean age was 76 years, 60% were men, and median left ventricular ejection fraction (LVEF) was 37%. Regarding the etiology of HF, ischemic cardiomyopathy comprised 24%. Patients with adverse events had higher age, higher prevalence of diabetes, chronic kidney disease, atrial fibrillation, ischemic cardiomyopathy etiology, ACE-Is or ARBs use, beta blockers use, diuretic use, statin use and intravenous inotropic use, higher brain natriuretic peptide, total bilirubin and CRP levels, and lower GNRI, heart rate, systolic blood pressure and eGFR, and lower hemoglobin, sodium, total cholesterol and albumin levels compared to those without adverse events (Tables 1 and 2).

**Table 1:** Baseline characteristics in patients with and without adverse events.

Variable	Overall (N = 685)	Events (N = 262)	No events (N = 423)	P-value
Age, years	76 ± 12	77 ± 11	75 ± 12	0.006
Male, n (%)	410 (60)	165 (63)	245 (58)	0.20
Body mass index	23.0 ± 4.3	22.5 ± 3.6	23.3 ± 4.6	0.016
GNRI	99.6 ± 11.2	97.1 ± 10.2	101.1 ± 11.5	<0.001
**Past history, n (%)**				
Hypertension	506 (74)	195 (74)	311 (74)	0.86
Diabetes	251 (37)	116 (44)	135 (32)	0.001
Dyslipidemia	361 (53)	143 (55)	218 (52)	0.48
CKD	363 (53)	183 (70)	180 (43)	<0.001
Hemodialysis	13 (2)	7 (3)	6 (1)	0.26
Prior MI	159 (23)	73 (28)	86 (20)	0.025
Atrial fibrillation	354 (52)	156 (60)	198 (47)	0.001
NYHA III or IV, n (%)	552 (81)	209 (80)	343 (81)	0.69
Heart rate, beat/min	92 ± 28	89 ± 25	94 ± 30	0.023
Systolic BP, mmHg	140 ± 32	133 ± 32	144 ± 31	<0.001
LVEF, %	37 (23,54)	35 (20,50)	39 (25,55)	0.15
**Etiology, n (%)**				
ICM	166 (24)	75 (29)	91 (22)	<0.001
DCM	119 (17)	37 (14)	82 (19)	
HCM	28 (4)	19 (7)	9 (2)	
HHD	70 (10)	19 (7)	51 (12)	
Valves	166 (24)	65 (25)	101 (24)	
**Oral medication on admission, n (%)**				
ACE-Is or ARBs	361 (53)	154 (59)	207 (49)	0.015
Beta blockers	358 (52)	162 (62)	196 (46)	<0.001
Diuretics	378 (55)	179 (68)	199 (47)	<0.001
Spironolactone	140 (20)	69 (26)	71 (17)	0.003
Statins	249 (36)	108 (41)	141 (33)	0.041

Continuous variables are presented as mean ± SD if normally distributed, and median (interquartile range) if not normally distributed. Categorical variables are presented as number of patients (%). ACE-I = angiotensin-converting enzyme inhibitor; ARB = angiotensin II receptor blocker; BP = blood pressure; CKD = chronic kidney disease; DCM = dilated cardiomyopathy; GNRI = geriatric nutritional risk index; HCM = hypertrophic cardiomyopathy; HHD = hypertensive heart disease; ICM = ischemic cardiomyopathy; LVEF = left ventricular ejection fraction; MI = myocardial infarction; NYHA = New York Heart Association.

**Table 2:** Findings of laboratory tests and intravenous treatment in patients with and without adverse events.

Variable	Overall (N = 685)	Events (N = 262)	No events (N = 423)	P-value
**Laboratory data on admission**				
Hemoglobin, g/dL	12.0 ± 2.1	11.5 ± 2.1	12.2 ± 2.1	<0.001
Sodium, mEq/L	140 (138,142)	139 (137,142)	141 (138,143)	<0.001
eGFR, ml/min	37.2 (25.1,49.0)	30.6 (20.2,40.5)	41.2 (28.8,53.5)	<0.001
BNP, pg/dL	605 (323,1114)	732 (393,1388)	551 (276,983)	0.001
hs TnT, ng/mL	0.04 (0.02,0.07)	0.05 (0.03,0.09)	0.03 (0.02,0.06)	0.73
Total bilirubin, mg/dL	0.3 (0.2,0.4)	0.3 (0.2, 0.5)	0.3 (0.2,0.4)	<0.001
Total cholesterol, mg/dL	157 ± 40	152 ± 41	161 ± 40	0.005
Albumin, g/dL	3.8 ± 0.4	3.7 ± 0.5	3.8 ± 0.4	<0.001
CRP, mg/dL	0.4 (0.1,1.2)	0.5 (0.2,2.1)	0.3 (0.1,0.9)	0.003
**Fatty acids concentration**				
EPA, μg/mL	40 (26,62)	39 (24,64)	40 (26,62)	0.71
DHA, μg/mL	108 (83,138)	101 (77,138)	111 (86,139)	0.018
AA, μg/mL	153 (123,182)	144 (113,169)	157 (129,188)	<0.001
DGLA, μg/mL	25 (18,33)	22 (17,30)	26 (20,35)	<0.001
N-3 PUFAs, μg/mL	151 (113,202)	146 (108,202)	154 (116,202)	0.11
N-6 PUFAs, μg/mL	178 (144,210)	166 (133,199)	187 (152,218)	<0.001
**Fatty acids ratio**				
EPA/AA	0.27 (0.18,0.40)	0.28 (0.18,0.45)	0.26 (0.17,0.37)	0.26
DHA/AA	0.72 (0.57,0.91)	0.73 (0.59,0.95)	0.71 (0.57,0.88)	0.11
N-3/N-6 PUFAs	0.86 (0.65,1.11)	0.88 (0.67,1.16)	0.84 (0.64,1.08)	0.14
**Intravenous treatment, n (%)**				
Diuretics	509 (75)	191 (74)	318 (76)	0.65
Vasodilators	412 (61)	149 (57)	263 (63)	0.13
Inotropes	94 (14)	54 (21)	40 (10)	<0.001

Continuous variables are presented as mean ± SD if normally distributed, and median (interquartile range) if not normally distributed. Categorical variables are presented as number of patients (%). AA = arachidonic acid; BNP = brain natriuretic peptide; CRP = C-reactive protein; DGLA = dihomo-gamma-linolenic acid; DHA = docosahexaenoic acid; eGFR = estimated glomerular filtration rate; EPA = eicosapentaenoic acid; hs TnT = high sensitive troponin T; N-3 = omega-3; N-6 = omega-6; PUFAs = polyunsaturated fatty acids.

**Fig 2:**
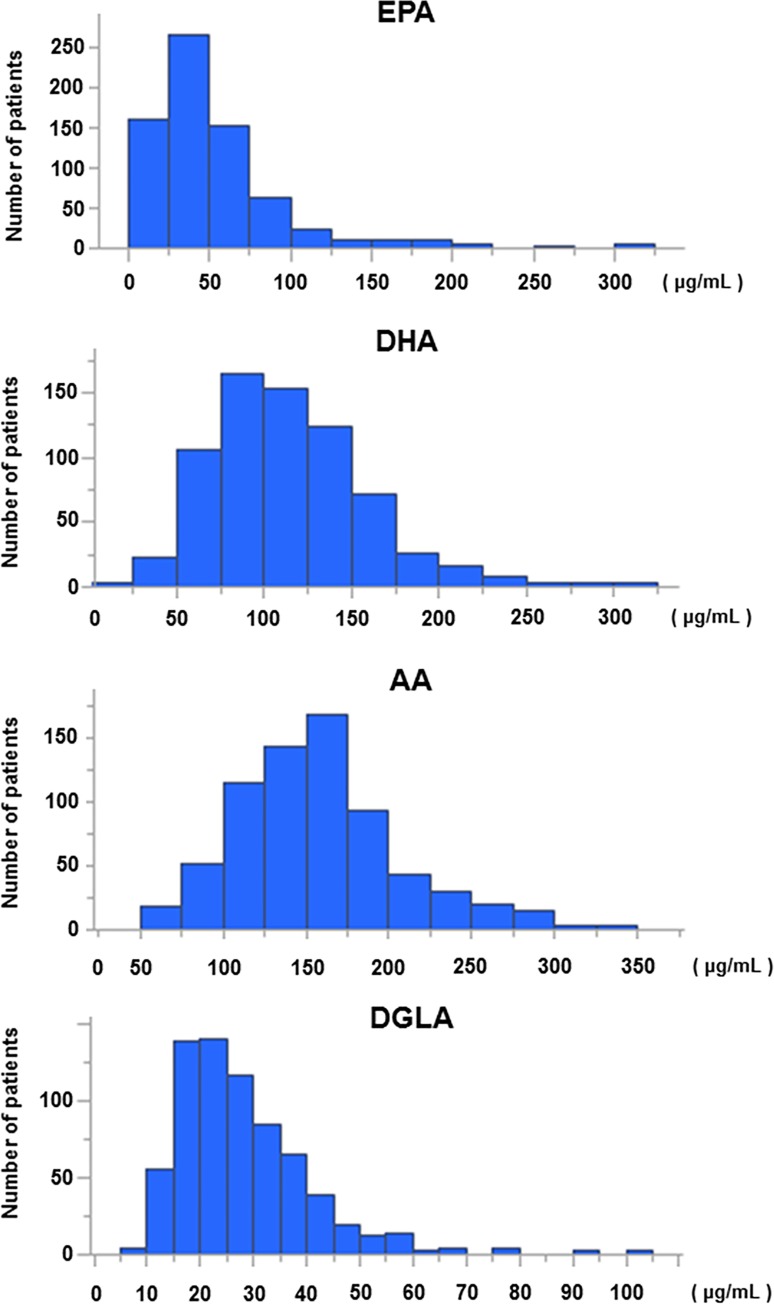
Distribution of PUFA levels. EPA, eicosapentaenoic acid; DHA, docosahexaenoic acid; AA, arachidonic acid; DGLA, dihomo-gamma linoleic acid.

The distribution of PUFA levels is shown in Fig 2; median PUFA (EPA, DHA, AA and DGLA) levels on admission were 40, 108, 153 and 25 μg/mL, respectively. Table 2 compares the levels and ratios of PUFAs in patients with and without adverse events. Although patients with adverse events had lower DHA, AA, DGLA, and n-6 (AA + DGLA) levels than those without, EPA and n-3 (EPA + DHA) levels, and EPA/AA, DHA/AA and n-3/n-6 ratios were comparable between the groups. In the present prospective cohort, n-6 rather than n-3 PUFA levels were associated with adverse events.

### Baseline Characteristics by Tertiles of N-6 PUFA Level

Baseline clinical characteristics are shown in Tables 3 and 4. Although patients with low n-6 PUFA level were older and had lower GNRI, systolic blood pressure, hemoglobin, eGFR, sodium, total cholesterol, albumin, EPA and DHA levels, and lower prevalence of statin use compared to those with high n-6 PUFA, there were no significant differences in New York Heart Association functional class, LVEF, etiology of HF and prevalence of hemodialysis among the tertiles.

**Table 3:** Baseline characteristics by tertiles of n-6 PUFA level.

Variable	Overall (N = 685)	Low (N = 228)	Intermediate (N = 226)	High (N = 231)	P-value
N-6 range, μg/mL	-	< 157	157–198	199 ≤	-
Age, years	76 ± 12	77 ± 11	76 ± 11	73 ± 13	0.008
Male, n (%)	410 (60)	151 (66)	134 (59)	125 (54)	0.029
Body mass index	23.0 ± 4.3	22.9 ± 4.2	22.9 ± 4.2	23.2 ± 4.4	0.67
GNRI	99.6 ± 11.2	97.5 ± 11.2	99.6 ± 10.4	101.6 ± 11.6	<0.001
**Past history, n (%)**					
Hypertension	506 (74)	161 (71)	166 (73)	179 (77)	0.24
Diabetes	251 (37)	92 (40)	69 (31)	90 (39)	0.059
Dyslipidemia	361 (53)	104 (46)	107 (47)	150 (65)	< 0.001
CKD	363 (53)	143 (63)	108 (48)	112 (49)	0.002
Hemodialysis	13 (2)	5 (2)	6 (3)	2 (1)	0.35
Prior MI	159 (23)	54 (24)	51 (23)	54 (23)	0.95
Atrial fibrillation	354 (52)	142 (62)	110 (49)	102 (44)	<0.001
NYHA III or IV, n (%)	552 (81)	190 (83)	180 (80)	182 (79)	0.42
Heart rate, beat/min	92 ± 28	86 ± 26	95 ± 28	95 ± 31	0.001
Systolic BP, mmHg	140 ± 32	133 ± 32	140 ± 30	146 ± 32	<0.001
LVEF, %	37 (23,54)	38 (23,55)	37 (23,52)	35 (22,54)	0.94
**Etiology, n (%)**					
ICM	166 (24)	57 (25)	52 (23)	57 (25)	0.73
DCM	119 (17)	34 (15)	43 (19)	42 (18)	
HCM	28 (4)	13 (6)	9 (4)	6 (3)	
HHD	70 (10)	21 (9)	20 (9)	29 (13)	
Valves	166 (24)	55 (24)	59 (26)	52 (23)	
**Oral medication on admission, n (%)**					
ACE-Is or ARBs	361 (53)	129 (57)	111 (49)	121 (53)	0.26
Beta blockers	358 (52)	128 (56)	114 (50)	116 (50)	0.37
Diuretics	378 (55)	152 (67)	120 (53)	106 (46)	<0.001
Spironolactone	140 (20)	54 (24)	43 (19)	43 (19)	0.34
Statins	249 (36)	67 (29)	76 (34)	106 (46)	< 0.001

Continuous variables are presented as mean ± SD if normally distributed, and median (interquartile range) if not normally distributed. Categorical variables are presented as number of patients (%). ACE-I = angiotensin-converting enzyme inhibitor; ARB = angiotensin II receptor blocker; BP = blood pressure; CKD = chronic kidney disease; DCM = dilated cardiomyopathy; GNRI = geriatric nutritional risk index; HCM = hypertrophic cardiomyopathy; HHD = hypertensive heart disease; ICM = ischemic cardiomyopathy; LVEF = left ventricular ejection fraction; MI = myocardial infarction; N-6 = omega-6; NYHA = New York Heart Association.

**Table 4:** Findings of laboratory tests and intravenous treatment by tertiles of n-6 PUFA level.

Variable	Overall (N = 685)	Low (N = 228)	Intermediate (N = 226)	High (N = 231)	P-value
N-6 range, μg/mL	-	< 157	157–198	199 ≤	-
**Laboratory data on admission**					
Hemoglobin, g/dL	12.0 ± 2.1	11.4 ± 2.2	11.9 ± 1.9	12.6 ± 2.1	<0.001
Sodium, mEq/L	140 (138,142)	139 (137,142)	140 (138,143)	140 (138,143)	<0.001
eGFR, ml/min	37.2 (25.1,49.0)	32.7 (21.9,44.8)	37.8 (27.6,51.5)	40.2 (27.3,51.3)	<0.001
BNP, pg/dL	605 (323,1114)	574 (323,1377)	663 (319,1154)	610 (337,944)	0.057
hs TnT, ng/mL	0.04 (0.02,0.07)	0.04 (0.03,0.07)	0.04 (0.02,0.07)	0.04 (0.02,0.06)	0.33
Total bilirubin, mg/dL	0.3 (0.2,0.4)	0.3 (0.2,0.6)	0.3 (0.2,0.4)	0.2 (0.2,0.3)	<0.001
Total cholesterol, mg/dL	157 ± 40	132 ± 29	159 ± 32	180 ± 43	<0.001
Albumin, g/dL	3.8 ± 0.4	3.6 ± 0.5	3.8 ± 0.4	3.9 ± 0.4	<0.001
CRP, mg/dL	0.4 (0.1,1.2)	0.5 (0.2,1.6)	0.5 (0.2,1.5)	0.3 (0.1,0.8)	0.11
AA, μg/mL	153 (123,182)	111 (95,123)	153 (144,160)	197 (181,231)	<0.001
DGLA, μg/mL	25 (18,33)	17 (15,22)	26 (20,31)	35 (27,42)	<0.001
EPA, μg/mL	40 (26,62)	29 (21,48)	43 (29,69)	48 (33,68)	<0.001
DHA, μg/mL	108 (83,138)	87 (67,109)	114 (89,139)	129 (98,158)	<0.001
Systolic BP, mmHg	140 ± 32	133 ± 32	140 ± 30	146 ± 32	<0.001
LVEF, %	37 (23,54)	38 (23,55)	37 (23,52)	35 (22,54)	0.94
**Intravenous treatment, n (%)**					
Diuretics	509 (75)	164 (72)	176 (79)	169 (73)	0.22
Vasodilators	412 (61)	120 (53)	134 (60)	158 (69)	0.002
Inotropes	94 (14)	34 (15)	31 (14)	29 (13)	0.73

Continuous variables are presented as mean ± SD if normally distributed, and median (interquartile range) if not normally distributed. Categorical variables are presented as number of patients (%). AA = arachidonic acid; BNP = brain natriuretic peptide; CRP = C-reactive protein; DGLA = dihomo-gamma-linolenic acid; DHA = docosahexaenoic acid; eGFR = estimated glomerular filtration rate; EPA = eicosapentaenoic acid; hs TnT = high sensitive troponin T; N-6 = omega-6.

### N-6 PUFA Level and Clinical Outcomes

**Fig 3:**
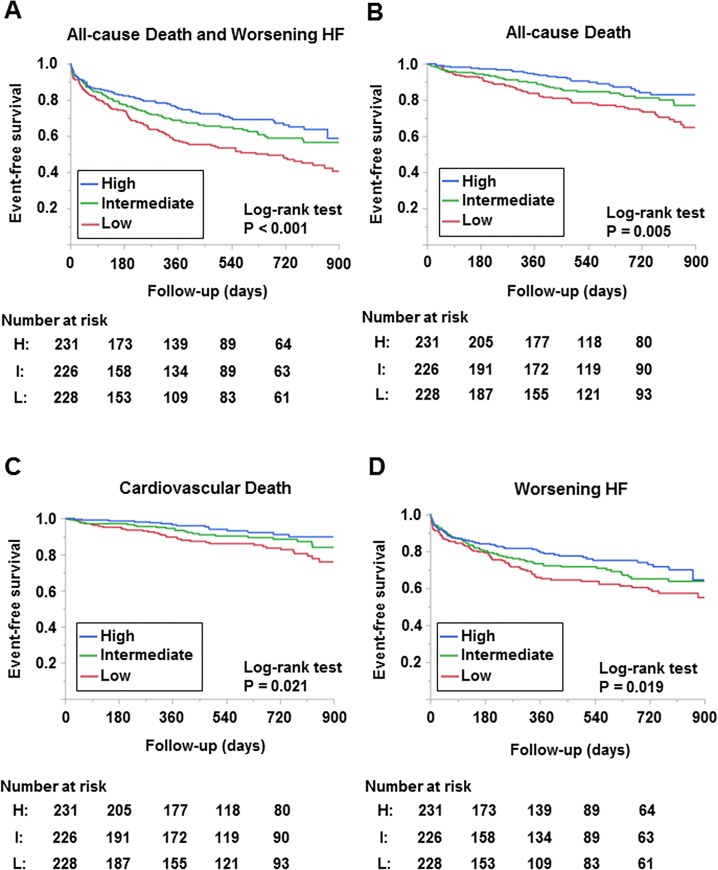
Kaplan-Meier analyses of clinical outcomes by n-6 PUFA level. Composite of all-cause death and worsening heart failure (A). All-cause death (B). Cardiovascular death (C), Worsening heart failure (D).

Kaplan-Meier analyses showed that lower total n-6 PUFA level on admission was significantly associated with worse clinical outcomes including the composite of all-cause death and worsening HF, all-cause death, cardiovascular death and worsening HF (log-rank; p < 0.001, p = 0.005, p = 0.021, p = 0.019, respectively, Fig 3). Cox proportional hazard analyses revealed that per point increase in n-6 PUFA level was associated with increased risk of adverse events [hazard ratio (HR): 0.994, 95% confidence interval (CI): 0.992–0.997, p < 0.001; HR: 0.995, 95% CI: 0.993–0.997, p < 0.001; and HR: 0.996, 95% CI: 0.993–0.999, p = 0.027; for models 1, 2, and 3, respectively], and patients with low n-6 PUFA level had a 1.4-fold increase in the risk of adverse events as compared with those with high n-6 PUFA level (p = 0.037, Table 5).

**Table 5:** Cox proportional hazards models for composite of death and worsening heart failure.

Variable	No. of events / at risk (%)	Model 1 Crude		Model 2 Age, sex-adjusted		Model 3 Fully-adjusted*	
**N-6 level as continuous variable**							
		**HR (95% CI)**	**P-value**	**HR (95% CI)**	**P-value**	**HR (95% CI)**	**P-value**
	262 / 685 (38.2%)	0.994(0.992–0.997)	<0.001	0.995(0.993–0.997)	<0.001	0.996(0.993–0.999)	0.027
**N-6 level as categorical variable**							
High	68 / 231 (29.4%)	1.00 (reference)		1.00 (reference)		1.00 (reference)	
Intermediate	82 / 226 (36.3%)	1.25 (0.90–1.73)	0.18	1.20 (0.87–1.66)	0.27	1.31 (0.93–1.83)	0.12
Low	112 / 228 (49.1%)	1.82(1.35–2.47)	<0.001	1.70(1.26–2.32)	<0.001	1.42(1.01–2.11)	0.037

*Model 3 was adjusted by age, sex, ICM etiology, systolic blood pressure, heart rate, estimated glomerular filtration rate, geriatric nutritional risk index, and hemoglobin, serum sodium, brain natriuretic peptide, total cholesterol, total bilirubin and C-reactive protein levels, and history of atrial fibrillation, dyslipidemia, diabetes and chronic kidney disease, and use of statins, diuretics, spironolactone, ACE-I or ARB, beta blockers and intravenous inotropes. ACE-I = angiotensin-converting enzyme inhibitor; ARB = angiotensin II receptor blocker; CI = confidence interval; HR = hazard ratio; ICM = ischemic cardiomyopathy.

## Discussion

The present study has shown that patients with lower n-6 PUFA level have a significantly higher incidence of adverse events (all-cause death and worsening HF) following ADHF, whereas n-3 PUFA level showed no significant association. Patients with lower n-6 PUFA level also had multiple co-morbid conditions including anemia and renal insufficiency. Furthermore, multivariate Cox analyses showed a lower n-6 PUFA level to be an independent determinant of adverse events. These findings suggest that n-6 rather than n-3 PUFA levels might be useful for identifying patients at high risk of adverse events following ADHF.

Although we could not explore the direct mechanisms for the association between lower n-6 PUFAs and worse clinical outcomes, there are several possible explanations. First, “consumption as a source of pro-inflammatory eicosanoids”; it has been strongly suggested that the two major types of long chain PUFAs, n-3 and n-6 PUFAs, have antagonistic effects. While the n-3 PUFAs emerged as cardioprotective, the n-6 PUFAs were shown to be pro-inflammatory [14, 15]. However, relevant intervention trials failed to link n-6 with a pro-inflammatory response [26–28]. Among them, Poudel-Tandukar et al. investigated the relationship between essential fatty acid status and CRP, and n-6 PUFAs were found to be inversely associated with serum CRP [28]. In the present study, n-6 PUFA levels also tended to be inversely associated with serum CRP level. N-6 PUFAs are known to be a major source of pro-inflammatory eicosanoids, and therefore it is speculated that n-6 PUFAs might be consumed by enhanced inflammatory responses in high risk ADHF patients. Second, “decreased production of vasodilators”; DGLA and AA are known to become several sources of respective different vasoactive eicosanoids. DGLA and AA are sources of prostaglandins (PG) of 1 series, and PG of 2 series, thromboxanes, leukotrienes of 4 series and nitric oxide (NO), respectively [29]. Among them, PGE_1_, which is formed from DGLA and is a potent arteriolar vasodilator, has beneficial effects on myocardial energetics and cardiac function in patients with severe ischemic HF [30]. PGI_2_ (prostacyclin), a product of AA, could have similar beneficial actions in view of the fact that PGI_2_ is also a potent vasodilator and possesses antiarrhythmic actions, similarly to PGE_1_ [31–34]. These PGs are known to enhance NO synthesis and release [35–38], suggesting that NO and PGs act in concert with each other to modulate cardiac function. They are also potent inhibitors of atherosclerosis, and interference with their action, as by free radicals, could lead to HF [39]. Taking these findings together, it is proposed that deficiency of n-6 PUFAs, especially DGLA and AA, may cause decreased production of PGE_1_, PGI_2_ and NO, thereby resulting in a decompensated state of HF. Third, “insufficiency due to malnutrition”; HF may promote malnutrition based on several mechanisms including gut edema, anorexia from inflammatory cytokine production, limitation in eating and food preparation due to fatigue, and increased work of breathing, especially in advanced right sided HF [40]. Despite the underlying mechanisms being unclear, insufficiency of n-6 PUFAs may occur more readily compared with that of n-3 PUFAs in advanced HF patients.

The effects of PUFAs as dietetic management in HF patients are controversial; therefore, little information is provided in the major HF management guidelines [41–43]. In spite of the fact that the Gruppo Italiano per lo Studio della Sopravvivenza nell'Infarto Miocardico-Heart Failure (GISSI-HF) trial showed a modest survival advantage from n-3 PUFA supplementation in HF [44], several recent clinical trials failed to show clear benefit from n-3 PUFAs in high risk patients with cardiovascular disease [17, 45]. In contrast, in animal models of congestive HF, higher dietary n-6 PUFA protected against the development of cardiac hypertrophy and systolic dysfunction, and improved total mortality [46, 47]; however, in patients with coronary artery disease, previous reports examining the effect of n-6 PUFA intake showed inconsistent results [48–50]. Adequately powered and well-designed randomized control trials to show the benefit of n-6 PUFAs are needed.

### Study Limitations

First, no information was available with regard to lifestyle and dietary intake of PUFAs; therefore, we could not exclude the influence of dietary intake on PUFA levels. Second, PUFAs were not measured in the cell membrane. PUFAs in the cell membrane were reported to be direct precursors of pro- and anti-inflammatory eicosanoids. However, it was reported that cell membrane EPA/AA ratio was strongly correlated with serum EPA/AA ratio in the Japanese population [51]. Third, we measured only EPA and DHA as n-3 PUFAs, and AA and DGLA as n-6 PUFAs among all PUFAs, although these four metabolites are representative precursors of eicosanoids that may affect the cardiovascular system. Finally, low n-6 PUFAs group were older and worse in their renal function; moreover, total bilirubin and cholesterol, albumin, and all fatty acids levels as well as GNRI were lower in the low n-6 PUFAs group. Therefore, low n-6 PUFAs level might reflect older chronic kidney disease with malnutrition.

## Conclusions

Lower n-6 PUFA level on admission was significantly related to worse clinical outcomes in ADHF patients, whereas n-3 PUFA level was not. These results suggest that measurement of circulating n-6 PUFAs rather than n-3 PUFAs levels on admission might provide information for risk stratification of ADHF patients.
